# The challenge of protracted measles outbreaks in Kismayo, Somalia: A mixed-method investigation of measles burden and vaccination coverage during a 2020–2021 outbreak

**DOI:** 10.1371/journal.pgph.0005143

**Published:** 2025-08-29

**Authors:** Douglas K. Lau, Simone Seebacher, Adan Abdi, Sugow Ahmed, Mohamed Bashir Nur, Iza Ciglenecki, Adrian Guadarrama, Etienne Gignoux

**Affiliations:** 1 Médecins Sans Frontières, Somalia Mission, Nairobi, Kenya; 2 Jubaland State Ministry of Health and Human Services, Kismayo, Somalia; 3 Médecins Sans Frontières, Operational Center Geneva, Geneva, Switzerland; 4 Epicentre, Paris, France; New York University Grossman School of Medicine, UNITED STATES OF AMERICA

## Abstract

There was a protracted measles outbreak in Kismayo, Somalia between 2020–2021. The outbreak persisted despite availability of measles containing vaccine (MCV) through Expanded Program on Immunization (EPI) services and reactive vaccination campaigns. We sought to estimate measles burden and MCV coverage during the outbreak while further identifying barriers and facilitators to care and vaccinations. We adopted a cross-sectional, sequential mixed-method approach with a retrospective household survey followed by key informant interviews (KIIs) and focus group discussions (FGDs). We used proxy-reported interview data from a household survey with a two-year recall period to estimate attack rates (ARs), case fatality ratios (CFRs), measles-specific mortality and MCV coverage. We performed thematic analysis on qualitative data from 12 KIIs and 8 FGDs. We surveyed 1,050 households representing 6,664 individuals and estimated an urban population of 405,181 (95%CI: 389,335–422,331). We identified 338 measles cases (AR: 5.1% [95%CI: 4.6-5.6]) and 11 measles deaths (CFR: 3.3% [95%CI: 1.4-5.2]). During the outbreak, we interpolated that 20,664 (95%CI: 17,909–21,651) measles cases and 682 (95%CI: 251–1230) deaths occurred across Kismayo. At start of recall, 49.5% (95%CI: 46.5-52.6) aged 6–59 months had one-or-more doses of MCV and this increased to 69.6% (95%CI: 66.9-72.2) by end of recall. Thematic analysis produced qualitative insights on *barriers to accessing medical care, barriers to routine vaccination through EPI, barriers to vaccination through mass campaigns* and *facilitating factors for care and vaccination*. We show an unacceptably high burden of measles due to limited access to medical care and low MCV coverage despite a widespread willingness to be vaccinated. To mitigate the problem of protracted outbreaks, we suggest adopting a consistent, community-centered approach to risk communication and community engagement, reducing non-healthcare costs associated with accessing care, ensuring daily availability of EPI vaccinations in all public facilities and overhauling the ways in which mass vaccination campaigns are implemented.

## Introduction

Measles is a highly contagious viral infection that can lead to serious complications or death, especially for vulnerable groups [1]. Case fatality ratios (CFRs) in well-resourced settings have been estimated at around 0.05% [2]; however, CFRs from low-resourced or emergency settings can be a great deal more severe. For instance, a 2019 average of CFRs from Sub-Saharan Africa was estimated to be 2.8%: a 5.6-fold increase over the previous estimate [3].

Measles containing vaccine (MCV) is part of the World Health Organization (WHO) Expanded Programme on Immunization (EPI). It involves a two-dose series that reduces symptom severity or prevents infection altogether. Vaccine effectiveness varies by context, but a recent review found that the first dose of measles vaccine (MCV1) given at 9–11 months of age was 85%-90% effective [4,5]. A second dose (MCV2) is recommended for all children to cover those without sufficient protection from the first [4,5]. It’s estimated that MCV coverage of 90%-95% is required for herd immunity [6].

In addition to the population health burden, childhood deaths from measles cause sizable economic losses that are further exacerbated in conflict-affected settings [7]. Moreover, MCV has a high return on investment, accounting for about 76% of the projected $21 in benefits for each dollar spent on immunization programmes in low- and middle-income countries [8]. Great advancements have been made globally in eliminating measles through vaccination. However, progress remains lacking in the African Region, with the Covid-19 pandemic further stalling routine immunization efforts [9,10].

### Somalia

Somalia had an estimated population of 18.4 million in 2023, with a 51% urban, 26% rural and 23% nomadic distribution [11,12]. Ongoing conflict, environmental disasters and difficult economic circumstances have led to considerable population displacement across the country. Access to public health services can be challenging and health facilities often rely on funding and technical support from local and international non-governmental organizations (NGOs) and United Nations (UN) agencies, including the UN Children’s Fund (UNICEF) and the WHO, among others [13,14]. In 2023, the infant mortality rate and the under 5 mortality rate were estimated at 67.8 and 104.0 deaths per 1000 live births, respectively [15].

**Fig 1:**
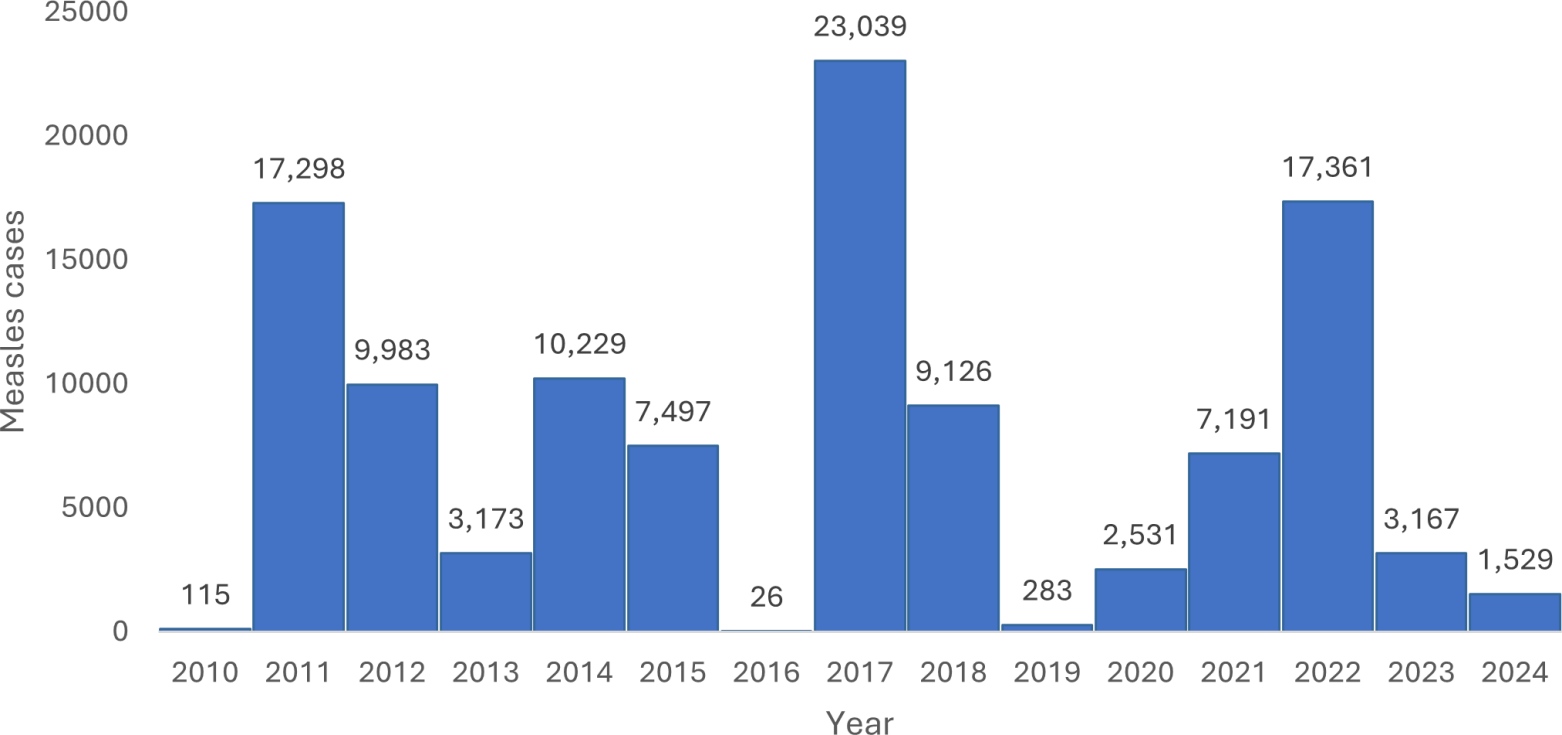
Country-wide measles cases reported from Somalia to the WHO (2010–2024).

Somalia has been facing intermittent outbreaks of measles over the past decade, with more than 111,019 cases reported to the WHO between 2010–2024 (see Fig 1) [16]. Despite this, there is a dearth of detailed studies on measles burden in this context, leaving a challenge for the quantification of population health impact in Somalia.

#### Vaccine access.

EPI services have been operating in Somalia since 1974, with free vaccinations for children under 2 years provided by the Ministry of Health and Human Services (MoH) [17]. Vaccine-preventable diseases targeted by the national immunization policy include tuberculosis, polio, diphtheria, pertussis, tetanus, hepatitis B, haemophilus influenza type B and measles (see Table 1). Measles vaccinations are scheduled for all children at 9 months (MCV1) and 15 months (MCV2) of age, by subcutaneous injection in the upper right arm with Vitamin A supplementation. MCV1 has been part of the routine schedule for many decades but MCV2 was more recently introduced in November 2021 [18].

**Table 1:** Routine immunization schedule for infants in Somalia, 0–15 months.

Age	Recommended Vaccines
At birth (up to 2 weeks)	BCG, OPV0
6 weeks	DTP-HepB + Hib1, OVP1
10 weeks	DTP-HepB + Hib2, OPV2
14 weeks	DTP-HepB + Hib3, OPV3, IPV1
9 months	Measles (MCV1), IPV2
15 months	Measles (MCV2)

EPI services should be available at all MoH health facilities at every point of contact with patients [17], though the logistics of service delivery depend on the operationalization the national policy at local levels. In practice, specific vaccinations like MCV may only be offered on select days of the week.

During outbreaks, the MoH can implement reactive vaccination campaigns as one-off strategies to bolster MCV coverage. When needs arise outside of acute outbreaks, Supplemental Immunization Activities (SIAs) are another type of mass vaccination campaign that can be implemented by the MoH. Typical target populations for these mass campaigns include children from 6 months to 5 years of age, with some exceptional campaigns including populations up to 10 or 15 years. Unlike routine EPI vaccinations – which are administered in health facilities – it is common practice, especially in Kismayo, for mass campaigns to employ mobile vaccination teams that administer doses through door-to-door household visits with cooler boxes and ice to preserve cold chain.

Despite the concerted efforts of the MoH and its partners, national immunization coverage for MCV1 remains low. The 2020 Somali Health and Demographic Survey estimated the national coverage of MCV1 among children aged 12–23 months at 22.7% overall, or 37.3% in urban settings – far below the requirements for herd immunity [12]. WHO and UNICEF estimates of national immunization coverage for the same age cohort remained at 46% for each year between 2010–2020, while official estimates from the Government of Somalia reported MCV1 coverage in the range of 45%-60% between 2016 and 2020 [18].

### Study setting: Kismayo, Somalia

Kismayo is a large port city in southern Somalia and the capital of Jubaland State. Jubaland State has lower vaccine coverage than the abovementioned national averages, with 2020 estimates of MCV1 coverage at 11.4% overall and 13.3% in urban settings [19]. Prior to the start of the outbreak in question, the most recent SIA for measles was conducted nationally in March 2018, with a target of 2.6 million children aged 6 months to 10 years in Somalia’s southern states, which includes Kismayo and the rest of Jubaland State. Unfortunately, no further details including campaign microplans or coverage achievements are available.

Kismayo hosts a large population of internal displaced people (IDPs) [20]. Its public health system is managed by the Jubaland State MoH. Kismayo has a network of public health centers known as maternal and child health (MCH) clinics that offer primary care, including EPI services. More specialized care is available at the regional referral facility, Kismayo General Hospital. The Jubaland State MoH also coordinates a network of humanitarian partners that collaborate on a variety of activities spanning from health service delivery and training to risk communication and community engagement (RCCE).

*Médecins Sans Frontières* (MSF) is an international humanitarian NGO that has been operating in Somalia since 1979, with a programmatic hiatus between 2013–2017. The *MSF Jubaland Project* ran from 2018-2023. Project activities included support to public health surveillance and emergency response across several districts of Jubaland State. Throughout the life of this project, MSF had no permanent operational presence in Kismayo or any other part of Jubaland State. Instead, team members used an *in-and-out approach* where staff based in Nairobi, Kenya would combine remote technical support with field visits for the implementation of project activities.

#### 2020–2021 measles outbreak in Kismayo.

**Fig 2:**
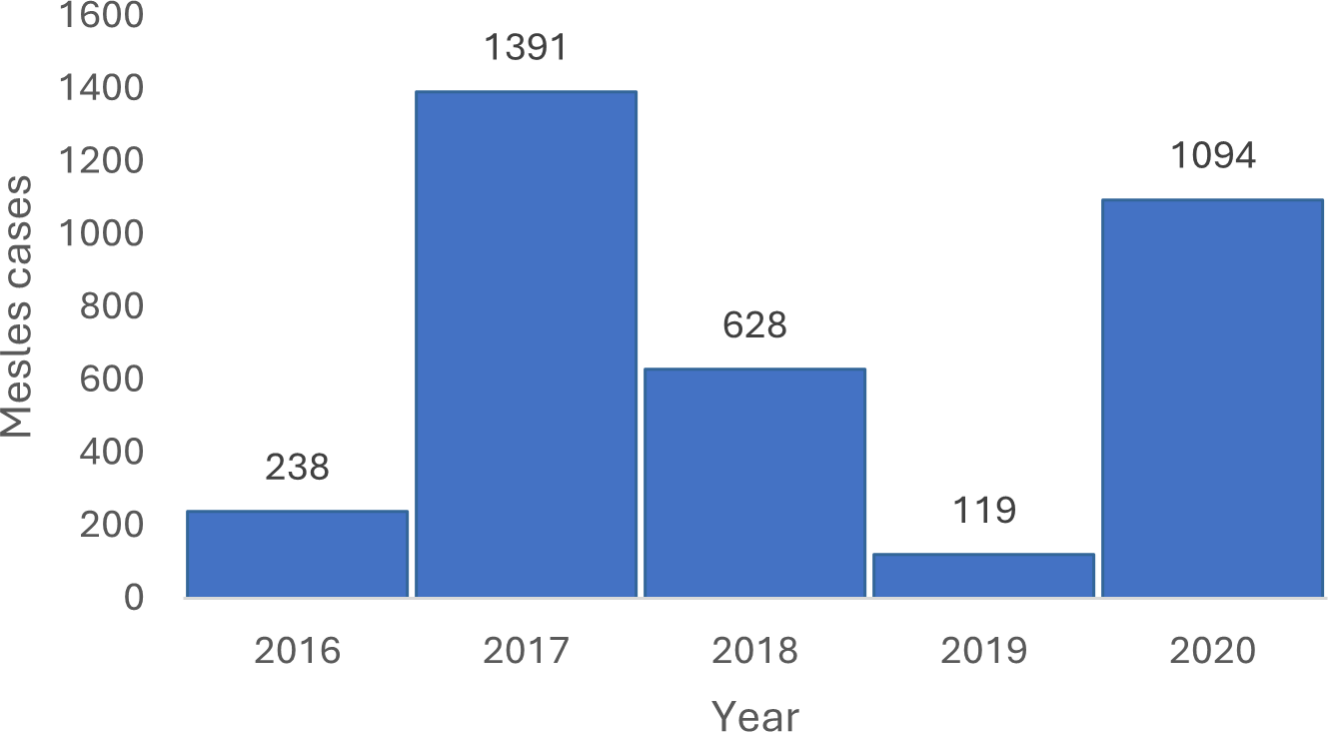
Measles cases seen at MoH facilities in Kismayo (2016–2020) [20].

In 2020, the MoH recorded an annual total of 1,094 suspected measles cases in Kismayo, which represented an 9-fold increase from the 119 cases recorded in 2019. This trend continued into 2021 with an annual total of 1,488 suspected cases (see Fig 2). Though testing capacity was limited, 33 of 57 samples (57.9%) sent for laboratory investigations were confirmed positive for measles [21].

During this protracted outbreak, the Jubaland State MoH requested interventional support from MSF. Due to the prevailing security conditions at the time, MSF was unable to initiate a direct medical intervention but instead agreed to support the MoH through collaboration on a joint operational research project to better understand the persistent nature of measles outbreaks that have afflicted Kismayo over the past several years.

Our overall objective was to estimate measles burden and MCV coverage in the urban areas of Kismayo during the 2020–2021 outbreak, while further identifying barriers and facilitators to care and vaccination. Specific objectives were to:

- Estimate the urban population size of Kismayo;
- Estimate the measles attack rate, case fatality ratio and measles-specific mortality during the recall period;
- Assess changes in MCV1 and MCV2 coverage over the recall period among children under 5 years and under 15 years; and
- To identify barriers and facilitators to measles care and vaccination.

## Materials and methods

Our study utilized a cross-sectional, sequential mixed-method approach with two phases of data collection. Phase one involved a retrospective household survey covering all urban areas of Kismayo. Preliminary findings then informed areas of inquiry for phase two: a qualitative investigation involving key informant interviews (KIIs) and focus group discussions (FGDs).

### Phase one: Household survey

#### Sampling procedures.

Sample size was calculated for measles-specific mortality using ENA for SMART (v.2020). We assumed a measles-specific mortality rate of 0.111 deaths per 10,000 population/day in alignment with a study from a similar setting [22]. We used an accuracy of 0.03, design effect of 1.0, recall period of 730 days, average household size of 6.6 [12], and a non-response rate of 10%. This yielded a minimum sample size of 1,093 households.

With technical support from the *MSF GIS Center* in Geneva, a satellite image with a spatial resolution of 0.4m was captured on 09/01/2021. Kismayo’s urban boundaries were demarcated and the rooftops of all structures were identified. Given the range of different housing options in Kismayo – from small informal structures to large multi-household apartment-style buildings – structures of all sizes were retained in the sampling frame. A simple random sample was conducted using QGIS (v3.22.11), with 1100 structures selected for inclusion. GPS coordinates for each sampled structure were superimposed on the satellite image and loaded into OsmAnd (v4.1) for enumerator team navigation.

#### Questionnaire design.

We used a structured questionnaire designed to capture data on a variety of topics including: household demographics, measles vaccination history, reasons for non-vaccination, measles morbidity, mortality, health-seeking behavior and reasons for not seeking care. We collected data with Kobo Collect (v2021.2.4) on mobile phones using a Somali language questionnaire, which was pre-tested and tailored prior to the start of the study.

#### Survey training and pilot study.

Twelve two-person, gender-balanced teams of survey enumerators participated in a three-day training and pilot study at a privately hired training venue in Kismayo. Six study supervisors, including three study authors, facilitated the training, in the Somali language, and provided on-the-ground supervision and troubleshooting support during data collection. The training covered topics including: study objectives and methods, ethical research practices and informed consent, team roles and responsibilities, questionnaire review and refinement, household navigation and procedures for data quality control.

As part of the training, a half-day pilot study was run with a subset of purposively selected households – distinct from those visited during the main study – within 2km of the training venue for practice with navigation, interviewing and electronic data collection. Pilot study data was rapidly analyzed and areas for improvement for each enumerator team were communicated before the main survey began. To strengthen data quality, this process of rapid analysis and review also continued for each day of data collection during the main survey.

#### Survey definitions.

The survey covered a two-year recall period: from 01/02/2020 to 31/01/2022. Self-identified heads of households acted as survey respondents. Households were defined as groups of people who “regularly ate meals together” (i.e., at least 3 shared meals per week over the past month).

Our measles case definition aligned with the standard WHO definition: as any case of fever, maculopapular rash and cough, coryza or conjunctivitis [23]. We did not include test results in our case definition due to limited access to testing in this setting. Measles case status was assessed verbally from the recall of respondents, followed by an additional set of questions that asked about symptoms consistent with the measles case definition (i.e., whether fever and rash along with cough, coryza or conjunctivitis were present). Cases that did not have reported symptoms consistent with the case definition were excluded.

We defined measles deaths as any death from suspected measles occurring within 28 days of symptom onset and was also based on the recall of respondents. We also asked a similar set of questions that confirmed symptoms prior to death. Any reported measles deaths that did not satisfy the criteria of symptoms consistent with the measles case definition and death within 28 days of symptom onset were excluded.

Measles vaccination status was assessed from the recall of respondents, for all household members between 6 months and 15 years of age at the start of the recall period. This was followed by visual inspections of vaccination cards, if available. In line with prevailing vaccination practices in Kismayo, the location where vaccinations were given was used as a proxy for whether the dose was received as part of routine EPI services (i.e., vaccinated in a health facility) or a reactive vaccination campaign (i.e., vaccinated at home). There were verbal reports of at least two reactive vaccination campaigns that happened in Kismayo during the recall period but the study authors were unable to obtain documentation of campaign dates or achievements. As such, we did not ask about specific campaigns and instead relied on the location of vaccination as the sole determinant of whether a dose was given through routine EPI or a reactive campaign. The Jubaland State MoH further confirmed that no SIAs took place in Kismayo during the recall period, so all home-based vaccinations were assumed to be part of reactive campaigns.

To support recall among respondents, enumerators had a set of printed photos depicting symptomatic measles patients and were electronically prompted in Kobo Collect to show these to the heads of household prior to asking questions on measles morbidity. For question on vaccinations, enumerators were prompted to remind survey respondents of the upper right arm site of injection and the standard dosing schedule of MCV at 9 and 15 months, respectively. They also prompted enumerators to request vaccination cards for visual inspection, whenever available.

To improve the recollection of dates, enumerators used a recall calendar that was tailored to locally relevant events over the two-year recall period. This included occasions like Ramadan, the *Gu* rains, the *Dayr* rains, and the start of Covid-19 public health measures in Kismayo. The questionnaire itself was programmed with constraint logic that restricted impossible answers, triggering pop-up alerts to encourage re-questioning for clarification. Constraint logic also prevented problems with missing data for most observations, as enumerators were required to submit a response for each question.

#### Data collection procedures.

Enumerator teams used OsmAnd on mobile phones to navigate to each sampled structure. Upon arrival, any sampled structure that was found to be non-residential in nature (e.g., shops, mosques), would be replaced with the “next nearest household” based on the fewest number of steps to the next front door. Structures were also replaced using the same method if they were not physically accessible (e.g., washed-out roads), or if a household was thought to possibly present a security threat to the research team. These judgements were made at the discretion of enumerator teams and recorded on the questionnaire form as additional data points.

If enumerator teams found that the structure had people living inside, they would find the head of household, introduce the study and ask for informed consent. Heads of households needed to be (1) present at the time of the visit and (2) at least 18 years of age to be eligible to participate. If teams failed to find an eligible head of household after 3 visits, the household was excluded without replacement. Any households with residents refusing informed consent were also excluded without replacement. In the case of a multi-household structure, enumerators were trained to take information from the first household they encountered, paying special care to the definition of a household described earlier. All survey communications were conducted in Somali and a Somali language information sheet was provided to all respondents.

#### Kismayo population estimate.

We estimated Kismayo’s population by counting the people sleeping under each randomly sampled roof the night before teams visited. Non-residential structures (e.g., shops, mosques) were counted as zeros, without replacement. For multi-household structures, this meant counting all people sleeping under the identified roof, regardless of whether they were in the same household or not. We calculated the average household size from all structures visited then multiplied this number by the total number of structures identified in our satellite image. Confidence interval estimation used a zero-inflated normal distribution, based on the assumption that many sampled buildings were non-residential in nature. Population estimates, along with under 5 proportions and IDP proportions were then used to calculate attack rates (ARs) and measles-specific mortality rates.

This method of counting household sizes for the estimation of urban population (i.e., without replacement of non-residential structures) was distinct from the method used for all other household demographic estimates. For the latter, non-residential structures were excluded, and the sample population was calculated instead using the residential households that replaced the non-residential structures, following the “next nearest household” method described above.

#### Statistical analysis.

Statistical analysis of survey data was performed using RStudio (v2024.12.0 + 467). 95% confidence intervals (95%CI) were calculated for all relevant estimates. Missing values were excluded using pairwise deletion in the calculation of specific rates.

ARs were calculated with the total number of verified measles cases divided by the age-specific population denominator from our population estimate. CFRs were calculated with the total number of measles deaths divided by the age-specific number of measles cases. Mortality rates per 10,000 population/day for the recall period were calculated as the number of deaths divided by total person-time. Household members who left, arrived, and were born during the recall period were considered as having contributed to half the recall period, assuming a constant mortality rate over time. For those who died, the exact time under observation was used. MCV coverage was calculated by taking the age-specific number of vaccinated children divided by the corresponding population estimate. Vaccination rates at the start and end of the recall period were compared.

### Phase 2: Qualitative investigations

KIIs using a semi-structured question guide were conducted with purposively sampled participants who had professional experience with measles care and vaccination in Kismayo. Key informants included frontline health care workers, MoH administrators and UN/NGO staff (see Table 2). Each KII lasted approximately 1 hour. All interviews were conducted virtually, in English, with one of two study authors responsible for qualitative data collection.

**Table 2:** Key informants.

Key Informant ID	Key Informant Classification	Interview Status
KII #1	Health Worker (MoH)	Included
KII #2	Health Worker (MoH)	Included
KII #3	Health Worker (MoH)	Included
KII #4	Health Worker (MoH)	Included
KII #5	Health Worker (MoH)	Excluded due to poor audio quality
KII #6	Administrator (MoH)	Included
KII #7	Administrator (MoH)	Included
KII #8	Administrator (MoH)	Included
KII #9	UN/NGO Staff	Included
KII #10	UN/NGO Staff	Included
KII #11	UN/NGO Staff	Included
KII #12	UN/NGO Staff	Included
KII #13	UN/NGO Staff	Included
**Key Informant Sex Distribution**		
Female: 3 (25.0%) Male: 9 (75.0%)		

FGDs were conducted with participants residing in Kismayo that were selected to represent a diverse range of community perspectives (see Table 3). Focus groups each included eight participants with groupings by participant category, IDP status and sex. Participant categories included: elders, religious leaders, traditional healers and community members (who did not fall into any other category). All FGDs were conducted in the Somali language, using a semi-structured discussion guide, with one facilitator and one notetaker, in a privately hired conference room. Each FGD lasted approximately 1 hour and transportation refunds were provided to all participants.

**Table 3:** FGD participants.

Group ID	Participants Category	Participants IDP Status	Participants Sex
FGD #1	Elders	non-IDP	Male
FGD #2	Elders	Mixed	Male
FGD #3	Religious Leaders	Mixed	Male
FGD #4	Traditional Healers	Mixed	Female
FGD #5	Traditional Healers	Mixed	Male
FGD #6	Community Members	non-IDP	Female
FGD #7	Community Members	IDP	Male
FGD #8	Community Members	IDP	Female
**Age Distribution**		**Sex Distribution**	
<35 years: 8 (12.5%) 35-55 years: 43 (67.2%) >55 years: 13 (20.3%)		Female: 24 (37.5%) Male: 40 (62.5%)	

For all qualitative investigations, audio-recordings were made with participant consent; otherwise, handwritten notes were used for analysis. Transcription and translation of audio recordings were carried out by a professional agency in Hargeisa, Somalia.

Thematic analysis was performed on English language transcripts and notes using a combination of Microsoft Word (Microsoft 365) and NVivo (v1.7) [24]. The analysis process followed a flexible, inductive-deductive approach, involving a combination of pre-determined themes on barriers and facilitators and data-driven subthemes to structure the analysis. Two authors independently analyzed the qualitative dataset before findings were harmonized. Due to contextual constraints that required long lead times for setting up qualitative investigations, we were not able to follow a conventional approach to rolling data collection until saturation was achieved. To mitigate this, we preemptively recruited a diverse and substantial number of key informants and FGD participants to ensure that we captured a broad range of perspectives and experiences.

### Ethical considerations

Prior to data collection, study sensitization meetings were held with local authorities and community leaders. Study participants were each given a Somali language information notice and had the opportunity to ask questions. Given ongoing security concerns, written consent forms were not used due to potential risks to participants from perceived affiliations with the MoH in case any forms got misplaced. Instead, verbal informed consent was obtained and recorded electronically on Kobo Collect.

Data collection for the household survey took place between 2–6 Feb 2022. During data collection, study team members had access to GPS coordinates of sampled households for navigation purposes and this could have led to identification of individual participants. However, these GPS data were deleted prior to analysis and no other identifying information was retained. Data collection for the KIIs took place between 24 Nov to 30 Dec 2022 and for the FGDs between 6 Dec to 12 Dec 2022. Participant names were removed from the qualitative datasets prior to analysis.

Ethical approval was obtained from the Médecins Sans Frontières (MSF) Ethical Review Board (#2175) and the Jubaland State MoH (MoH/JSS/DG/060/2021). Activities were conducted in accordance with the International Ethical Guidelines for Biomedical Research Involving Human Subjects and the International Ethical Guidelines for Epidemiological Studies [25,26].

## Results

### Study population and characteristics

Enumerator teams visited 1,103 structures over six days of data collection. 9.8% of these structures were non-residential, inaccessible, or “high-risk” structures that were replaced. 1,050 households provided informed consent, with 53 (4.8%) refusals. This yielded a sample population of 6,664 individuals. Of these, 50.1% were female, 19.1% were under 5 years of age and 27.4% identified as IDPs (see S1 Fig). The average household size was 6.04 overall, or 5.76 for IDP households and 6.12 for non-IDP households.

### Kismayo population estimate

Based on 66,358 structures identified by satellite image and an average of 6.08 individuals sleeping under each rooftop initially visited by enumerator teams, the urban population of Kismayo at the time of data collection was estimated to be 405,181 (95%CI: 389,335–422,331).

### Measles burden

#### Measles cases and deaths.

Households reported 367 cases of measles over the two-year recall period. Of these, 338 (92.1%) reported symptoms that met our case definition and were retained for analysis (see Table 4).

**Table 4:** Measles cases, MCV status and care-seeking behavior.

IDP Status	n	Age Category	Sex	MCV Status	Sought Health Care	Care Seeking Delay*	Reason for Not Seeking Care**
**Overall**	338	<5y: 153 (45.3%) 5y-14y: 113 (33.4%) >=15y: 72 (21.3%)	F: 177 (52.4%) M: 161 (47.6%)	1 Dose: 141 (41.7%) 2 Dose: 7 (2.1%) Never vaccinated: 134 (39.6%) Don’t know: 56 (16.6%)	Yes: 150 (44.4%) No: 188 (55.6%)	< 1 week: 101 (53.7%) >1 week: 13 (6.9%) Don’t know: 36 (19.1%)	Mild disease: 73 (38.8%) Belief care not effective: 65 (34.6%) Distance: 24 (12.8%) Social stigma: 4 (2.1%) Caretaker busy: 3 (1.6%) Cost: 2 (1.1%) Security concerns: 2 (1.1%) Don’t know: 15 (8.0%)
**Non-IDP**	217	<5: 92 (42.4%) 5y-14y: 71 (32.7%) >=15y: 54 (24.9%)	F: 108 (49.8%) M: 109 (50.2%)	1 Dose: 85 (39.2%) 2 Dose: 6 (2.8%) Never vaccinated: 84 (38.7%) Don’t know: 42 (19.4%)	Yes: 85 (39.2%) No: 132 (60.8%)	< 1 week: 61 (71.8%) >1 week: 3 (3.5%) Don’t know: 21 (24.7%)	Mild disease: 54 (40.9%) Belief care not effective: 42 (31.8%) Distance: 11 (8.3%) Social stigma: 4 (3.0%) Caretaker busy: 3 (2.3%) Cost: 2 (1.5%) Security concerns: 1 (0.8%) Don’t know: 15 (11.4%)
**IDP**	121	<5: 61 (50.4%) 5y-14y: 42 (34.7%) >=15y: 18 (14.9%)	F: 69 (57.0%) M: 52 (43.0%)	1 Dose: 56 (46.3%) 2 Dose: 1 (0.8%) Never vaccinated: 50 (41.3%) Don’t know: 14 (11.6%)	Yes: 65 (53.7%) No: 56 (46.3%)	< 1 week: 40 (61.5%) >1 week: 10 (15.4%) Don’t know: 15 (23.1%)	Mild disease: 19 (33.9%) Belief care not effective: 23 (41.1%) Distance: 13 (23.2%) Social stigma: 0 Caretaker busy: 0 Cost: 0 Security concerns: 1 (1.8%) Don’t know: 0

*Among those seeking care.

**Among those not seeking care.

190 all-cause deaths were reported, with 8.9% (n = 17) having suspected measles as the proxy-reported cause of death. 11 deaths from suspected measles (64.7%) were reported to have occurred within 28 days of symptom onset and were retained for analysis. Of these 11 measles deaths, three (27.3%) had a history of MCV1, none had a history of MCV2, six (54.5%) sought health care prior to death but only three (27.3%) did so within seven days of symptom onset (see Table 5). Of the six measles deaths that were excluded, three deaths occurred beyond 28 days of symptom onset – at 61, 92 and 304 days. Another thee of the excluded deaths didn’t provide enough data to determine time between symptom onset and death.

**Table 5:** Measles deaths*, MCV status and care-seeking behavior.

#	IDP Status	Age	Sex	MCV Status	Sought Health Care	Care Seeking Delay	Reason for Not Seeking Care
1	IDP	9m	Female	Never vaccinated	No	–	Distance
2	IDP	1y	Female	1 Dose	Yes	> 1 week	–
3	IDP	1y	Female	Never vaccinated	No	–	Distance
4	IDP	1y	Male	Never vaccinated	Yes	> 1 week	–
5	IDP	3y	Female	Never vaccinated	No	–	Distance
6	IDP	4y	Female	1 Dose	Yes	< 1 week	–
7	IDP	4y	Female	1 Dose	Yes	< 1 week	–
8	IDP	12y	Male	Never vaccinated	No	–	Security
9	Non-IDP	1y	Female	Never vaccinated	Yes	Don’t know	–
10	Non-IDP	4y	Male	Never vaccinated	Yes	< 1 week	–
11	Non-IDP	22y	Male	Don’t know	Don’t know	–	–

*Deaths occurring within 28 days of symptom onset.

#### Reasons for not seeking care.

Among the measles cases we identified, only 44.4% (n = 150/338) sought health care. The most common reasons for foregoing care included perceptions that “measles is a mild disease” (38.8%), “medical care is not effective against measles” (34.6%) and the far distances to the nearest facility (12.8%). In comparison, reasons related to security and social stigma were rarely reported (see Table 4).

#### Attack rate, case fatality ratio and measles-specific mortality.

Among those meeting the case definition, we found an overall AR of 5.1% (95%CI: 4.6-5.6). Children under 5 had an AR of 12.0% (95%CI: 10.2-13.8), while children under 5 from IDP households had an AR of 17.0% (95%CI: 13.1-20.9).

Measles deaths occurring within 28 days of symptom onset made up 5.8% of all deaths (n = 11), yielding an overall CFR of 3.3% (95%CI: 1.4-5.2). Children under 5 had a CFR of 5.9% (95%CI: 2.1-9.6), while children under 5 from IDP households had a CFR of 11.5% (95%CI: 3.4-19.5).

The overall crude mortality rate (CMR) was 0.39 deaths per 10,000 population/day (95%CI: 0.34-0.45). The under 5 mortality rate (U5MR) was 0.57 deaths per 10,000 population/day (95%CI: 0.39-0.74). The overall measles-specific mortality rate was 0.02 deaths per 10,000 population/day (95%CI: 0.01-0.04). Children under 5 had a measles-specific mortality of 0.12 deaths per 10,000 population/day (95%CI: 0.04-0.21), while children under 5 from IDP households had a rate of 0.33 deaths per 10,000 population/day (95%CI: 0.09-0.58). See Table 6 for a summary of ARs, CFRs, and mortality estimates.

**Table 6:** Attack rates, case fatality ratios and measles-specific mortality rates, by age and IDP status.

	non-IDP	IDP	Overall
Attack Rate (measles cases*/ Kismayo population estimate)			
All ages	4.5% (95%CI: 3.9-5.1)	6.6% (95%CI: 5.5-7.8)	5.1% (95%CI: 4.6-5.6)
Under 5 years	10.0% (95%CI: 8.1-12.0)	17.0% (95%CI: 13.1-20.9)	12.0% (95%CI: 10.2-13.8)
Case Fatality Ratio (measles deaths**/ measles cases*)			
All ages	1.4% (95%CI:0-2.9)	6.6% (95%CI:2.1-11.1)	3.3% (95%CI:1.4-5.2)
Under 5 years	2.2% (95%CI:0-5.2)	11.5% (95%CI:3.4-19.5)	5.9% (95%CI:2.1-9.6)
Measles-specific Mortality Rate (measles deaths per 10,000 population/day)***			
All ages	0.01 (95%CI: -0.01-0.02)	0.06 (95%CI:0.02-0.11)	0.02 (95%CI:0.01-0.04)
Under 5 years	0.04 (95%CI: -0.02-0.09)	0.33 (95%CI:0.09-0.58)	0.12 (95%CI:0.04-0.21)
All-cause Mortality Rate (any cause deaths per 10,000 population/day)***			
CMR	0.36 (95%CI: 0.34-0.45)	0.48 (95%CI: 0.29-0.42)	0.39 (95%CI: 0.34-0.45)
U5MR	0.41 (95%CI: 0.23-0.58)	0.95 (95%CI: 0.54-1.36)	0.57 (95%CI: 0.39-0.74)

* Measles cases with reported symptoms meeting the measles case definition.

** Deaths among measles cases occurring within 28 days of symptom onset.

*** Calculated using our population estimate for Kismayo.

### MCV coverage

At the start of the recall period, 49.5% (95%CI:46.552.6) of children aged 6m-59m had one or more doses of MCV (see Table 7). This later increased to 69.6% (95%CI: 66.9-72.2), resulting in a 20.1% growth in coverage over the two-year recall period. Looking at the wider age group of 6m-15y, we start with 48.8% (95%CI: 46.9-50.7) having one or more doses and end with 61.7% (95%CI: 59.5-63.4), giving a more modest increase of 12.9% over the two years in question. For additional data on the 6m-15y population, see S1 Table.

**Table 7:** MCV coverage, by age and IDP status.

IDP Status	Age	Doses	% Coverage at Recall Start (n) [95%CI]	% Coverage at Recall End (n) [95%CI]	*% Increased Coverage Over Recall Period (n)*	% Vacc. Card Available (n)*	% Vaccinated through EPI (n)*	% Vaccinated through Campaign (n)*
**Overall**	**6m to 59m n(start)=1028 n(end)=1157**	1	46.1% (474) [95%CI: 43.1-49.2]	61.3% (709) [95%CI: 58.5-64.1]	15.2% (235)	69.3% (491)	94.6% (671)	4.8% (34)
		2	3.4% (35) [95%CI:2.3-4.5]	8.3% (96) [95%CI: 6.7-9.9]	4.9% (61)	64.6% (62)	86.5% (83)	12.5% (12)
		>=1	49.5% (509) [95%CI: 46.5-52.6]	69.6% (805) [95%CI: 66.9-72.2]	20.1% (296)	68.7% (553)	93.7% (754)	5.7% (46)
**Non-IDP**	**6m to 59m** **n(start)=712** **n(end)=821**	1	49.4% (352) [95%CI: 45.8-53.1]	62.6% (514) [95%CI: 59.3-65.9]	13.2% (162)	71.2% (371)	96.3% (495)	2.9% (15)
		2	3.5% (25) [95%CI: 2.2-4.9]	8.4% (69) [95%CI: 6.5-10.3]	4.9% (44)	62.3% (43)	89.9% (62)	8.7% (6)
		>=1	52.9% (377) [95%CI: 49.3-56.6]	71.0% (583) [95%CI: 67.9-74.1]	18.1% (206)	71.0% (414)	95.5% (557)	3.6% (21)
**IDP**	**6m to 59m** **n(start)=316** **n(end)=336**	1	38.6% (122) [95%CI: 33.2-44.0]	58.0% (195) [95%CI: 52.8-63.3]	19.4% (73)	61.5% (120)	90.3% (176)	9.7% (19)
		2	3.2% (10) [95%CI: 1.2-5.1]	8.0% (27) [95%CI: 5.1-10.9]	4.8% (17)	70.4% (19)	77.8% (21)	22.2% (6)
		>=1	41.8% (132) [95%CI: 36.3-47.2]	66.1% (222) [95%CI: 61.0-71.1]	24.3% (90)	62.6% (139)	88.7% (197)	11.3% (25)

*Based on population receiving at least one dose at end of recall period.

**Based on all MCV doses received by end of recall period.

MCV2 coverage among those 6m-59m started at 3.4% (95%CI: 2.3-4.5) and increased to 8.3% (95%CI: 6.7-9.9), yielding a 4.9% increase over the recall period. For those 6m-15y, MCV2 coverage started at 3.4% (95%CI: 2.8-4.1) and later increased to 6.3% (95%CI: 5.4-7.1).

Among children 6m-59m, MCV received through EPI accounted for 93.7% of all doses received during the recall period. Conversely, only 5.7% of MCV doses received were attributed to door-to-door reactive campaigns. Vaccine cards were available and visually inspected for 68.7% of children aged 6m-59m.

#### Reasons for non-vaccination.

Among the 1426 individuals of vaccine-eligible age who were never vaccinated, no vaccine being offered (41.4%) and far distances to the nearest health facility (22.9%) were most commonly reported. Reasons related to lack of trust in “western vaccines” (4.5%) and the belief that the measles vaccine is ineffective (3.9%) were least often reported (see Table 8).

**Table 8:** Reasons for non-vaccination among population of vaccine-eligible age, by IDP status.

Reasons for non-vaccination	*non-IDP,* *6m-15y [n = 906)*	*IDP,* *6m-15y [n = 515)*	*Overall,* *6m-15y [n = 1426)*
No vaccine offered (facility or door-to-door)	32.9% (298)	56.7% (292)	41.4% (590)
Distance to facility too far	21.1% (191)	25.8% (133)	22.9% (327)
Don’t trust western vaccines	6.2% (56)	1.6% (8)	4.5% (64)
Belief vaccine ineffective	5.7% (52)	0.6% (3)	3.9% (55)
Fear of side effects	2.3% (21)	4.5% (23)	3.1% (44)
Not eligible age at time of facility visit	3.3% (30)	2.5% (13)	3.0% (43)
Security situation prevented vaccination	3.4% (31)	1.6% (8)	2.7% (39)
All other reasons (combined)	2.8% (25)	2.3% (12)	2.6% (37)
Don’t know/ Refuse	22.3% (202)	4.5% (23)	15.9% (227)

### Estimate of measles cases and deaths during recall period

Based on the AR, CFR and urban population size, we interpolated that during the 2020–2021 outbreak, there were a total of 20,664 (95%CI 17,909–23,651) measles cases and 682 (95%CI: 251–1230) measles deaths that occurred in Kismayo (see Table 9).

**Table 9:** Estimated measles cases and deaths during outbreak in Kismayo.

Population	Estimated Cases	Estimated Deaths
Overall, all ages	20,664 (95%CI: 17,909–23,651)	682 (95%CI: 251–1230)

### Qualitative findings

13 KIIs were conducted with five MoH health workers, three MoH administrators and five UN or NGO staff (see Table 2). One KII with a MoH health worker was excluded from analysis due to poor audio quality. Data from 12 KIIs were retained for analysis. Eight FGDs were conducted with eight participants each, totalling 64 participants. Two groups included IDPs, two groups included non-IDPs, and four groups included participants with mixed IDP status (see Table 3). Although participants were not specifically selected based on whether they were past or present caregivers of children of vaccine-eligible age, discussions indicated a strong representation of caregivers within our sample.

We coded data under the pre-conceived themes of *barriers to accessing measles care, barriers to routine vaccination through EPI, barriers to vaccination through mass campaigns* and *facilitators for care and vaccination*. Each theme also had several subthemes that were deduced from the data (see Table 10).

**Table 10:** Themes and sub-themes identified from thematic analysis.

Themes	Sub-Themes
1. Barriers to accessing measles care	Measles knowledge gaps Traditional healing, costly access to facilities and delays to care
2. Barriers to routine vaccination through EPI	Community perspectives on measles vaccination Missed opportunities for routine vaccinations
3. Barriers to vaccination through mass campaigns	Human resource challenges Inadequate RCCE
4. Facilitators for care and vaccination	A historically rooted familiarity with measles in the community Widespread willingness to access medical care and vaccination Support from local leaders and trusted community members Well-distributed health facilities and strong partnership network

Perspectives on measles care and vaccination differed at times between FGD participants and key informants, however, a variety common concepts were also expressed throughout. As data collection progressed, findings began to converge toward saturation, supporting the robustness of insights from across the dataset. Many of our qualitative findings complemented findings from the household survey regarding reasons for not seeking care and for non-vaccination.

### Theme 1: Barriers to accessing measles care

#### Measles knowledge gaps.

FGD participants described a long history of lived experiences with measles and measles vaccination in the community and highlighted the passing down of knowledge from earlier generations as a key method of learning.

*“Our parents taught us the signs of the disease, and we now recognize it based on the symptoms they taught us.”*

*- FGD#2, Clan Elders*

*“I have a personal connection to measles, as it has claimed the lives of several of my family members … Even though I was young, I have heard stories about the devastating effects of the disease.”*

*- FGD#6, Community Members*

FGD participants agreed that care for sick children was a shared responsibility between mother and father, though each fulfilled different roles in the provision of care. Mothers were seen as responsible for recognizing symptoms and taking initial steps to access care while fathers were seen as final decision-makers in the household.

Many FGD participants demonstrated knowledge of measles symptoms, potential complications like blindness, and the risk of death, especially among children. However, despite this overall familiarity, FGD participants and key informants alike highlighted gaps in knowledge among parts of the community who lacked access to information, especially among IDPs.

*“I was not previously aware of measles, but I have learned about it through personal experience. One of my sons died … due to a lack of knowledge about the disease... I did not take him to the hospital when he became sick because I did not recognize the symptoms”*

*- FGD#8, Community Members*

Besides those who lacked access to information, participants also spoke about smaller segments of the community that did not accept health messages due to mistrust, sometimes related to misinformation and rumors. There was some disagreement over how prevalent these opinions were, but participants reported that opportunities for open discussion, especially with the involvement of trusted community members, could lead to improved acceptance of health messages.

#### Traditional healing, costly access to facilities and delays to care.

Descriptions of traditional healing methods included a wide variety of practices including the use of local herbs, goat’s milk and the blood of slaughtered animals. Participants also frequently mentioned Qur’anic recitations as an important practice to heal the sick.

FGD participants and key informants alike described a commonly used hybrid approach to measles treatment, where caregivers first went to traditional healers before turning to hospital care if symptoms persisted. While opinions varied on the effectiveness of traditional healing methods, there was broad consensus — even among many traditional healers — that severe cases would benefit from hospital care.

*“If traditional treatment and Quranic recitation do not bring about any improvement, then the patient is taken to the hospital for medical treatment.”*

*- FGD#5, Traditional Healers*

When attempting to seek care, participants highlighted substantial barriers to access, including distance, cost and competing household priorities. Participants reported that only diagnosis and referrals for measles patients were offered at MCH facilities, making Kismayo General Hospital the only viable public treatment option. FGD participants and key informants alike recognized that travel costs placed a heavy burden on households, especially in situations requiring overnight hospitalization. This was further intensified for caregivers of multiple children, who would have to make special arrangements for the children left at home during hospital visits.

*“Sometimes we come across severe cases of measles and when we suggest hospital admission… the caretakers refuse to stay due to the lack of food and financial resources. Some others may say ‘I have 4 children in my house, and I do daily work as a house builder. I struggle to make ends meet and cannot afford to admit the baby.’”*

*-Key Informant #3, MoH Health Worker*

Given these challenges, FGD participants across several groups reported that caregivers commonly resorted to traditional healers, who were more easily accessible in the community and offered treatments that could be administered at home.

### Theme 2: Barriers to routine vaccination through EPI.

#### Community perspectives on measles vaccination.

Across FGDs, there was clear consensus that Islamic teachings do not restrict access to measles vaccination in Somalia. The vast majority of FGD participants viewed the measles vaccine as an effective means of prevention, with many – including traditional healers – sharing stories of vaccinated children suffering less during episodes of sickness compared to unvaccinated children from the same household.

*“I have seen the difference between my vaccinated and unvaccinated children”*

*- FGD#3, Religious Leaders*

*“Some of my children had severe measles, and some felt like it was just a small fever. My first child was unvaccinated. He contracted measles while being unvaccinated. I took him to so many hospitals to treat him.”*

*- FDG#4, Traditional Healers*

In addition to reported challenges with access involving distance and cost, FGD participants and key informants agreed that some members of the community avoided vaccination for reasons including a commonly held fear of needles, fear of side effects following vaccination and confusion over breakthrough infections. Challenges with vaccine acceptance was seen to be especially pronounced among IDP households and the frequent arrival of new IDPs from insecure, remote or otherwise difficult-to-reach areas with limited access to vaccination was commonly identified as a key contributing factor in ongoing measles transmission. Mistrust in vaccine quality and a perceived lack of vaccine effectiveness was also a reported concern for some members of the community, with apprehension over expired doses, problems with cold chain or suspicion of procurement channels.

*“IDPs, especially those who are newly displaced … may have limited knowledge about measles outbreaks, which makes it harder for them to access and accept [vaccination] services.”*

*-Key Informant #10, UN/NGO Staff*

*“Some individuals in urban areas also refuse to vaccinate their children due to lack of awareness or misinformation, such as concerns over the composition, quality, and date of the vaccine.”*

*- FGD#1, Clan Elders*

#### Missed opportunities for routine vaccinations.

Despite widespread reports on the well-distributed network of MCH facilities with dedicated staff for EPI, FGD participants and key informants alike commonly highlighted challenges with the once-a-week offerings of MCV in these facilities. Participants shared stories of vaccine-eligible children being brought to facilities only to find that the vaccines they needed weren’t being offered that day. This translated into missed opportunities for vaccination and uncertainty over whether children would complete scheduled vaccinations during future visits.

*“The [MCH facilities] give measles vaccinations only on one day, either Saturday or Sunday... This is challenging for people because if a mother comes on a day when the vaccine is not being administered, she won’t be able to get it and may not be able to come back another day”*

*-Key Informant #2, MoH Health Worker*

FGD participants and key informants also raised issues with intermittent vaccine supply shortages, knowledge gaps related to MCV2, and opportunities for further expansion of facilities to cover underserved parts of Kismayo, especially for IDPs.

### Theme 3: Barriers to vaccination through mass campaigns

#### Human resource challenges.

Many FGD participants and key informants alike voiced mistrust over the technical capacity of the staff hired to administer door-to-door vaccinations and this reportedly impacted vaccine uptake among community members.

*“One major challenge is a lack of trust in the qualifications of the workers”*

*- FGD#3, Religious Leaders*

*“The health workers who administer the vaccinations often lack sufficient knowledge about the disease”*

*-Key Informant #3, MoH Health Worker*

This criticism echoed concerns raised by MoH administrators, who highlighted widespread challenges in recruiting sufficient human resources. They emphasized the need to assemble large teams – often involving hundreds of vaccinators – to reach sizable populations during short, 5-day long campaign durations. Some FGD participants agreed that campaigns of this length would be insufficient in meeting the needs of such a large urban center but challenges with funding meant that short-term campaigns were the only feasible option.

*“When we are selecting vaccinators, we have to choose healthcare providers who are trained in vaccine delivery… it can be challenging to find 200 or more healthcare providers who have the time to do this.”*

*-Key Informant #11, MoH Administrator*

*“I had raised concerns about the recent vaccination campaign and recommended to the ministry that a 5 day campaign for measles vaccination would not be sufficient for Kismayo City. However, the NGO responsible for the campaign explained that they only had funding for 5 days.”*

*- FGD#3, Religious Leaders*

Human resource challenges for reactive campaigns also extended to the supervision of campaign activities, where many key informants raised questions over accountability for campaign staff. MoH administrators acknowledge the challenges with supervision but noted that budgetary constraints limited their ability to address the issue.

*“Many teams were created and told to do mass campaigns, but there were too few team leaders to supervise them... Strict supervision and close monitoring are necessary in mass campaigns to ensure that the team is doing the right thing and vaccinating the correct individuals.”*

*-Key Informant #10, UN/NGO Staff*

*“We don’t have enough resources to monitor effectively. Because in Kismayo, we can only have a maximum of 10 supervisors for the whole city, which has a population of half a million people. It is challenging to monitor the vaccinators with such a limited number of supervisors.”*

*-Key Informant #13, MoH Administrator*

#### Inadequate RCCE.

FGD participants and key informants widely reported insufficient RCCE efforts before, during and in-between planned campaigns. A key informant who previously worked as a campaign vaccinator described the need to spend extended periods of time at each household conducting ad-hoc sensitization sessions, without appropriate training or technical support on RCCE.

Participants reported that campaign teams also lacked involvement of trusted community members, especially in IDP communities. This lack of local representation was perceived to negatively impact community trust, ultimately undermining vaccine uptake.

*“If someone is employed in a neighborhood where they do not reside, they may find it challenging to connect with and give awareness to the community as they lack knowledge of the area and its residents.”*

*- FGD#8, Community Members*

*“Having a respected figure like [a religious leader] speak to people about vaccination and be present while people are being vaccinated can have a positive impact.”*

*-FGD#3, Religious Leaders*

UN/NGO informants further noted the lack of vaccine coverage surveys and weaknesses with other types of monitoring and evaluation, as this meant that campaign achievements couldn’t be routinely verified and further added to challenges with the coordination of response efforts.

### Theme 4: Facilitators for care and vaccination

#### A historically rooted familiarity with measles in the community.

Across FGDs and KIIs, it was clear that there was an abundance of lived experiences with measles, with many stories of community members benefitting from medical care and vaccination. These experiences could form the basis of a strengthened RCCE strategy with more widely accepted, locally relevant content to improve community awareness of outbreak control priorities.

#### Widespread willingness to access medical care and vaccination.

Despite the presence of some degree of mistrust in the community, we understood that many community members wanted to access health services but were blocked by barriers related to distance, cost and competing household priorities. Well-funded programmatic efforts to reducing these barriers could result in substantial improvements to the uptake of medical care and vaccination services.

#### Support from local leaders and trusted community members.

FGD participants and key informants alike consistently emphasized that RCCE activities needed to more actively involve local leaders and other trusted community members. Leveraging this type of support – especially during acute outbreaks – could enhance community trust and improved utilization of services.

#### Well-distributed health facilities and strong partnership network.

The decentralized MCH facilities in Kismayo were widely recognized as useful community resources and Kismayo General Hospital was further acknowledged as an essential care option for severe cases. KIIs also revealed a strong network of humanitarian partners working toward measles outbreak management. Although some areas for improvement related to supply and management were identified, these health system components represent key strengths that should be leveraged toward future outbreak control priorities.

## Discussion

This study represents the first mixed-method research on measles burden and MCV coverage in Somalia. Our results provide a unique, data-driven set of estimates that describe the damaging impact that measles had on the Kismayo population. We further offer some insights into *why* protracted outbreaks have persisted and further contribute to a growing literature on challenges with vaccination in Somalia [27–29].

Though largely consistent with studies from similar settings [3,30,31], our estimates of measles burden are unquestionably worrying. With an attack rate of 12.0% (95%CI: 10.2-13.8) for children under 5 and 17.0% (95%CI: 13.1-20.9) for children under 5 from IDP households, we see that the outbreak spread rapidly through the Kismayo population. CFRs of 5.9% (95%CI: 2.1-9.6) for children under 5 and 11.5% (95%CI: 3.4-19.5) for children under 5 from IDP households along with our estimate of 682 (95%CI: 251–1230) measles deaths occurring over the recall period further highlight the outbreak’s deadly impact.

Deaths from measles occur from complications that can develop during the disease course. Timely medical treatment is an effective means to prevent complications and ultimately avert mortality [1,5,6,32]. Despite this, less than half (n = 150/338) of our measles cases sought medical care. Among the 11 measles deaths we recorded, six (54.5%) accessed a health facility prior to death but only three (27.3%) did so within seven days of symptom onset.

Malnutrition is a well-established risk factor for measles mortality [1,5]. A Standardized Monitoring and Assessment of Relief and Transitions (SMART) survey conducted halfway through our study recall period found a global acute malnutrition (GAM) rate of 34.0% among children aged 6–59 months in the Lower Juba Region, which includes Kismayo [33]. Although we lack patient-level data on nutritional status, the high prevalence of malnutrition in the population may have contributed to the notable degree of measles mortality in our study.

Our qualitative findings showed that barriers to accessing care involved a combination of knowledge gaps on the importance of timely treatment and logistical barriers related to distance, cost and competing household priorities which may have led to some degree of compromise for more easily accessible traditional healing options. Taken together, these factors highlight the importance of future efforts to increase demand for services with a strengthened approach to RCCE, while also improving access by reducing non-healthcare costs incurred by those seeking care.

Our findings explain ongoing measles transmission by means of low MCV coverage, reportedly exacerbated by inflows of IDPs from areas without easy access to vaccination. By the end of the recall period, an MCV coverage 69.6% (95%CI: 66.9-72.2) among children 6–59 months was achieved. While these estimates are a great improvement from those previously reported [19], the coverage in Kismayo remains well below the 90–95% target threshold for herd immunity [6]. Notably, MCV coverage among children up to 15 years of age was found to be similar to those under 5 years; however, as this older age group is no longer eligible for routine EPI services, it will be crucial to include them in future catch-up campaigns that explicitly target expanded age cohorts. We also observed relatively low coverage of MCV2, which is consistent with expectations given that much of the recall period occurred prior to its introduction in Somalia. Strengthening the rollout of MCV2 will be essential for future efforts toward enhancing measles outbreak control.

From the household survey, the most reported reason for non-vaccination was that vaccines were not offered, either during health facility visits or door-to-door campaigns. This is consistent with our qualitative findings, which showed a generally positive attitude towards measles vaccination that was often shaped by beneficial experiences with vaccination within households or the wider community. Despite numerous studies reporting vaccine hesitancy among Somali communities living abroad [34–37], mistrust of vaccines didn’t appear to be a widespread barrier to vaccination in this context. This was reinforced by qualitative findings suggesting that community members who hold mistrust toward vaccines are likely open to reconsidering their position if opportunities for RCCE involving trusted local leaders are established.

In terms of EPI services, we believe that the limited schedule of facility-based MCV offerings represents a major operational shortcoming, especially during an acute outbreak scenario. Going forward, it should be an important priority to increase vaccine supply and funding so that facility-based vaccination can happen on a daily basis – at every point of contact with patients – in line with both the national immunization policy [17] and WHO recommendations on reducing missed opportunities for vaccination [38].

During the outbreak, reactive vaccination campaigns only accounted for about 5.7% of all doses received. These findings are difficult to interpret, as Covid-19 public health restrictions may have limited the scale and scope of campaign activities and campaign reports documenting the initially planned campaign targets were not available for review. However, with so few households benefitting from door-to-door vaccination – which is standard practice for mass vaccination campaigns in Kismayo and the rest of Jubaland State – there is still considerable opportunity to enhance the impact of future campaigns with extended campaign durations, more comprehensively trained personnel, improved supervision and more consistent, community-centered RCCE efforts across all phases of planned campaigns.

### Strengths and limitations

Our study benefited from a robust random sampling method, which included a novel GIS-enabled approach to population size estimation. This in turn provided reliable population denominators for the estimation of rates. The survey’s large quantitative dataset was strengthened by comprehensive training and close supervision during data collection. Qualitative investigations benefitted from remote and in-person access to diverse stakeholders and enabled the triangulation of findings across data sources.

Several important limitations should also be acknowledged. Our household survey’s two-year recall period was relatively long and may have increased the risk of recall bias. Our identification of measles cases and deaths was based on respondents’ recollections rather than medical records, which could have further contributed to reporting inaccuracies. We attempted to mitigate these challenges by using recall calendars, photo references and verbal confirmation of measles symptoms. The consistency of our epidemiological rates with other studies [3,31,32] also suggests that any residual bias was likely limited.

Three suspected measles-related deaths were excluded from the analysis due to missing symptom onset dates. If these deaths occurred within 28 days of symptom onset, their exclusion may have led to an underestimation of CFR and measles-specific mortality.

There was also risk of confusion between MCV and other EPI vaccinations. However, enumerators were trained to highlight the typical site of vaccination (i.e., upper right arm) and the high proportion of available vaccination cards likely improved data accuracy. We also included children aged 6–8 months in our coverage analysis, recognizing that they would have only been eligible for measles vaccination through mass campaigns, not routine EPI services. Lack of access to vaccination campaign reports also prevented us from comparing our findings with campaign targets, limiting our ability to assess the achievements of these campaigns. We also could not directly assess objective measures of vaccine availability, vaccine delivery methods, or cold chain challenges.

For our qualitative investigations, some degree of selection bias may have occurred due to reliance on local leaders to recruit participants. MSF’s involvement with the study may also have introduced response bias, as awareness of MSF’s prior operational presence in the region could have led to the tailoring of responses to encourage more MSF-supported interventions. However, similarities in responses across FGDs and KIIs alleviate some of these concerns.

Finally, our findings have limited generalizability to non-urban parts of Somalia due to contextual differences in population dynamics, healthcare access, and security conditions. However, there are variety of urban or semi-urban contexts in Somalia, where our findings are likely to have greater external validity.

## Conclusion

We have shown an unacceptably high burden of measles in Kismayo, both in terms of morbidity and mortality. Households are often familiar with the disease but forego medical care due to gaps in knowledge or barriers related to insurmountable distances, costs or competing household priorities. Despite what appears to be a common willingness to be vaccinated, MCV coverage remains below the threshold needed to prevent community transmission, fueled in part by limited EPI offerings and operational shortcomings in the implementation of mass campaigns.

These are challenging circumstances in the complicated context of Somalia but there are some achievable measures that could improve the situation in the near term. These include efforts to bolster measles knowledge through consistent, community-centered approaches to RCCE, reducing non-healthcare costs associated with accessing care, ensuring daily availability of EPI vaccinations in all MoH facilities and overhauling the ways in which mass vaccination campaigns are carried out. By taking strategic action, we hope that stakeholders in health can blunt the burden of measles and safeguard the health of communities in Kismayo and beyond.

## Supporting information

S1 Checklist STROBE checklist for cross-sectional studies. (PDF)

S2 Checklist Inclusivity in global research. (DOCX)

S1 Fig Age and sex distribution of household survey population. (PDF)

S1 Table Full table of MCV coverage, by age and IDP status. (PDF)
