# Supplementary material for: The challenge of protracted measles outbreaks in Kismayo, Somalia: A mixed-method investigation of measles burden and vaccination coverage during a 2020–2021 outbreak
Source: PLOS Glob Public Health. 2025 Aug 29;5(8):e0005143. doi: 10.1371/journal.pgph.0005143 (PMC12396683; doi:10.1371/journal.pgph.0005143)
Supplement: S1 Checklist — (PDF) [file pgph.0005143.s001.pdf]

S1 Checklist. STROBE checklist for cross-sectional studies

	Item No	Recommendation	Line Numbers
**Title and abstract**	1	(*a*) Indicate the study's design with a commonly used term in the title or the abstract	Title
		(*b*) Provide in the abstract an informative and balanced summary of what was done and what was found	Abstract
**Introduction**			
Background/ rationale	2	Explain the scientific background and rationale for the investigation being reported	1-100
Objectives	3	State specific objectives, including any prespecified hypotheses	92-100
**Methods**			
Study design	4	Present key elements of study design early in the paper	102-286
Setting	5	Describe the setting, locations, and relevant dates, including periods of recruitment, exposure, follow-up, and data collection	62-80; 108-240
Participants	6	(*a*) Give the eligibility criteria, and the sources and methods of selection of participants	145-226
Variables	7	Clearly define all outcomes, exposures, predictors, potential confounders, and effect modifiers. Give diagnostic criteria, if applicable	145-226;
Data sources/ measurement	8*	For each variable of interest, give sources of data and details of methods of assessment (measurement). Describe comparability of assessment methods if there is more than one group	145 - 226
Bias	9	Describe any efforts to address potential sources of bias	670-708
Study size	10	Explain how the study size was arrived at	109-114
Quantitative variables	11	Explain how quantitative variables were handled in the analyses. If applicable, describe which groupings were chosen and why	227-240
Statistical methods	12	(*a*) Describe all statistical methods, including those used to control for confounding	108-240
		(*b*) Describe any methods used to examine subgroups and interactions	Not relevant
		(*c*) Explain how missing data were addressed	184-185
		(*d*) If applicable, describe analytical methods taking account of sampling strategy	Not relevant
		(*e*) Describe any sensitivity analyses	Not relevant

**Results**			
Participants	13*	(a) Report numbers of individuals at each stage of study—eg numbers potentially eligible, examined for eligibility, confirmed eligible, included in the study, completing follow-up, and analysed	288-295
		(b) Give reasons for non-participation at each stage	288-295
		(c) Consider use of a flow diagram	Not relevant
Descriptive data	14*	(a) Give characteristics of study participants (eg demographic, clinical, social) and information on exposures and potential confounders	288-295
		(b) Indicate number of participants with missing data for each variable of interest	Not relevant
Outcome data	15*	Report numbers of outcome events or summary measures	309-386
Main results	16	(*a*) Give unadjusted estimates and, if applicable, confounder-adjusted estimates and their precision (eg, 95% confidence interval). Make clear which confounders were adjusted for and why they were included	309-386
		(*b*) Report category boundaries when continuous variables were categorized	Not relevant
		(*c*) If relevant, consider translating estimates of relative risk into absolute risk for a meaningful time period	Not relevant
Other analyses	17	Report other analyses done—eg analyses of subgroups and interactions, and sensitivity analyses	Not relevant
**Discussion**			
Key results	18	Summarise key results with reference to study objectives	607-669
Limitations	19	Discuss limitations of the study, taking into account sources of potential bias or imprecision. Discuss both direction and magnitude of any potential bias	670-708
Interpretation	20	Give a cautious overall interpretation of results considering objectives, limitations, multiplicity of analyses, results from similar studies, and other relevant evidence	607-669
Generalisability	21	Discuss the generalisability (external validity) of the study results	705-708
**Other information**			
Funding	22	Give the source of funding and the role of the funders for the present study and, if applicable, for the original study on which the present article is based	Funding Declaration

*Give information separately for exposed and unexposed groups.

Note: An Explanation and Elaboration article discusses each checklist item and gives methodological background and published examples of transparent reporting. The STROBE checklist is best used in conjunction with this article (freely available on the Web sites of PLoS Medicine at http://www.plosmedicine.org/, Annals of Internal Medicine at http://www.annals.org/, and Epidemiology at http://www.epidem.com/). Information on the STROBE Initiative is available at www.strobe-statement.org.
