# Supplementary figures and images for: The challenge of protracted measles outbreaks in Kismayo, Somalia: A mixed-method investigation of measles burden and vaccination coverage during a 2020–2021 outbreak
Source: PLOS Glob Public Health. 2025 Aug 29;5(8):e0005143. doi: 10.1371/journal.pgph.0005143 (PMC12396683; doi:10.1371/journal.pgph.0005143)

**S2 Fig. Age and sex distribution of household survey population**

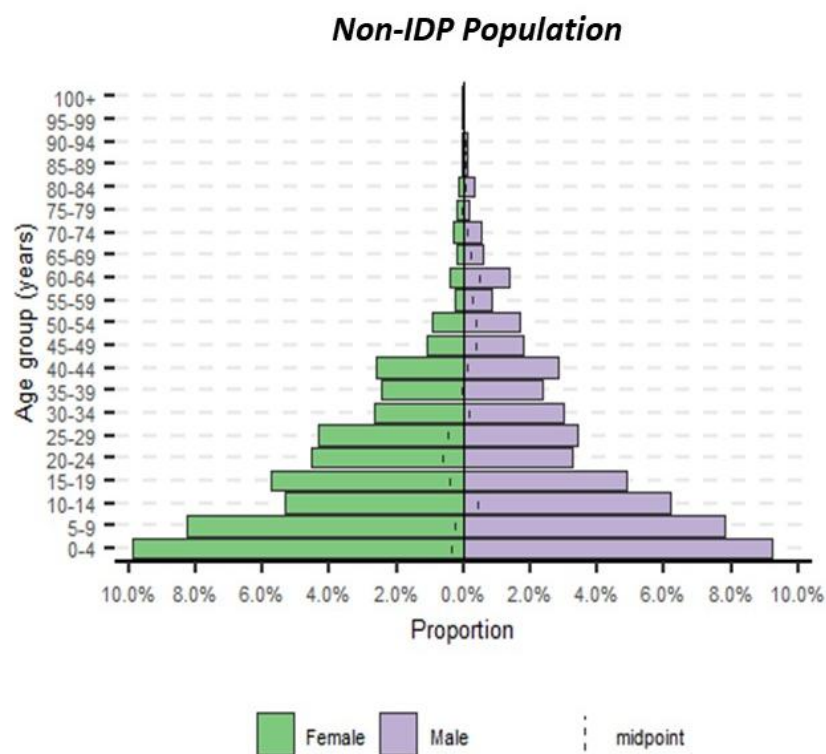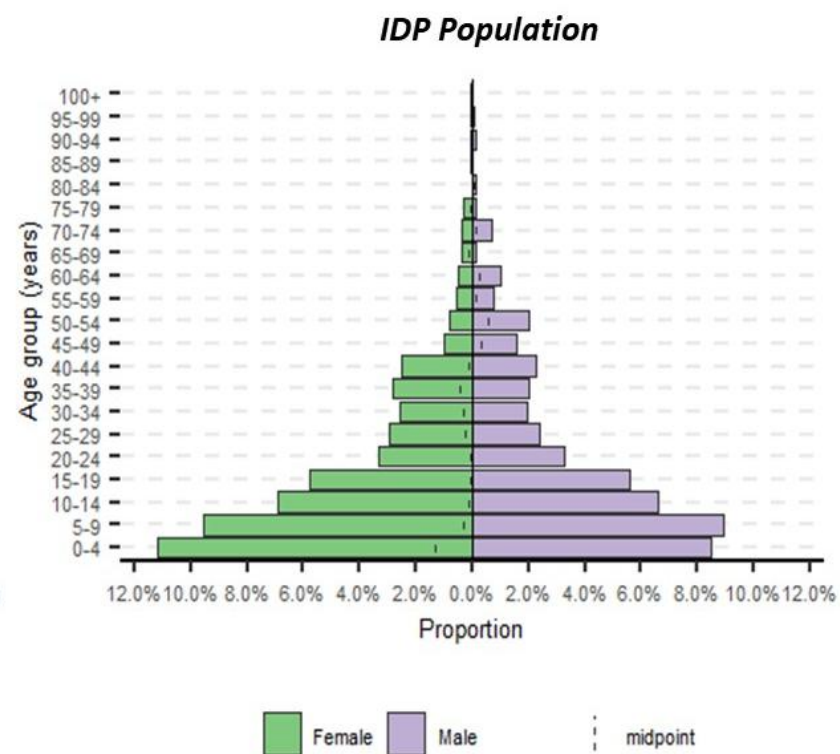

Supplement: S1 Fig — (PDF) [file pgph.0005143.s003.pdf]
