# Supplementary material for: The challenge of protracted measles outbreaks in Kismayo, Somalia: A mixed-method investigation of measles burden and vaccination coverage during a 2020–2021 outbreak
Source: PLOS Glob Public Health. 2025 Aug 29;5(8):e0005143. doi: 10.1371/journal.pgph.0005143 (PMC12396683; doi:10.1371/journal.pgph.0005143)
Supplement: S1 Table — (PDF) [file pgph.0005143.s004.pdf]

S3 Table. Full table of MCV coverage, by age and IDP status

IDP Status	Age	Doses	% Coverage at Recall Start [95%CI] (n)	% Coverage at Recall End [95%CI] (n)	*% Increased Coverage Over Recall Period (n)*	*% Vacc. Card Available (n)**	*% EPI Vacc. (n)**	*% Campaign Vacc. (n)**
**Overall**	**6m to 59m n(start)=1028, n(end)=1157**	1	46.1% (474) [95%CI: 43.1-49.2]	61.3% (709) [95%CI: 58.5-64.1]	15.2% (235)	69.3% (491)	94.6% (671)	4.8% (34)
		2	3.4% (35) [95%CI:2.3-4.5]	8.3% (96) [95%CI: 6.7-9.9]	4.9% (61)	64.6% (62)	86.5% (83)	12.5% (12)
		>=1	49.5% (509) [95%CI: 46.5-52.6]	69.6% (805) [95%CI: 66.9-72.2]	20.1% (296)	68.7% (553)	93.7% (754)	5.7% (46)
**Overall**	**6m to 15y n(start)=2734 n(end)=3075**	1	45.4% (1240) [95%CI: 43.5-47.2]	55.4% (1703) [95%CI: 53.2-56.7]	10% (463)	57.5% (979)	92.7% (1579)	6.8% (115)
		2	3.4% (94) [95%CI: 2.8-4.1]	6.3% (193) [95%CI: 5.4-7.1]	2.9% (99)	65.3% (126)	85.5% (165)	13.0% (25)
		>=1	48.8% (1334) [95%CI: 46.9-50.7]	61.7% (1896) [95%CI: 59.9-63.4]	12.9% (562)	58.3% (1105)	92.0% (1744)	7.4% (140)
**Non-IDP**	**6m to 59m n(start)=712 n(end)=821**	1	49.4% (352) [95%CI: 45.8-53.1]	62.6% (514) [95%CI: 59.3-65.9]	13.2% (162)	71.2% (371)	96.3% (495)	2.9% (15)
		2	3.5% (25) [95%CI: 2.2-4.9]	8.4% (69) [95%CI: 6.5-10.3]	4.9% (44)	62.3% (43)	89.9% (62)	8.7% (6)
		>=1	52.9% (377) [95%CI: 49.3-56.6]	71.0% (583) [95%CI: 67.9-74.1]	18.1% (206)	71.0% (414)	95.5% (557)	3.6% (21)
**Non-IDP**	**6m to 15y n(start)=1878 n(end)=2144**	1	49.6% (931) [95%CI: 47.3-51.8]	59.0% (1264) [95%CI: 56.9-61.0]	9.4% (333)	64.0 (809)	94.3% (1192)	5.0% (63)
		2	3.1% (58) [95%CI: 2.3-3.9]	6.2 (134) [95%CI: 5.2-7.3]	3.1% (76)	69.4% (93)	88.8% (119)	9.0% (12)
		>=1	52.7% (989) [95%CI: 50.4-54.9]	65.2% (1398) [95%CI: 63.2-67.2]	12.5% (409)	64.5% (902)	93.8% (1311)	5.4% (75)
**IDP**	**6m to 59m n(start)=316 n(end)=336**	1	38.6% (122) [95%CI: 33.2-44.0]	58.0% (195) [95%CI: 52.8-63.3]	19.4% (73)	61.5% (120)	90.3% (176)	9.7% (19)
		2	3.2% (10) [95%CI: 1.2-5.1]	8.0% (27) [95%CI: 5.1-10.9]	4.8% (17)	70.4% (19)	77.8% (21)	22.2% (6)
		>=1	41.8% (132) [95%CI: 36.3-47.2]	66.1% (222) [95%CI: 61.0-71.1]	24.3% (90)	62.6% (139)	88.7% (197)	11.3% (25)
**IDP**	**6m to 15y n(start)=845 n(end)=921**	1	36.2% (306) [95%CI: 33.0-39.5]	47.3% (436) [95%CI: 44.1-50.6]	11.1% (130)	39.0% (170)	88.1% (384)	11.9% (52)
		2	3.7% (31) [95%CI: 2.4-4.9]	5.9% (54) [95%CI: 4.3-7.4]	2.2% (23)	55.6% (30)	79.6% (43)	20.4% (11)
		>=1	39.9% (337) [95%CI: 36.6-43.2]	53.2% (490) [95%CI: 50.0-56.4]	13.3% (153)	40.8% (200)	87.1% (427)	12.9% (63)
